# CA 19-9 and CA 125 as potential predictors of disease recurrence in resectable lung adenocarcinoma

**DOI:** 10.1371/journal.pone.0186284

**Published:** 2017-10-19

**Authors:** Sofi Isaksson, Per Jönsson, Nastaran Monsef, Hans Brunnström, Pär-Ola Bendahl, Mats Jönsson, Johan Staaf, Maria Planck

**Affiliations:** 1 Division of Oncology and Pathology, Department of Clinical Sciences Lund, Lund University, Lund, Sweden; 2 Department of Thoracic Surgery, Lund University, Skåne University Hospital, Lund, Sweden; 3 Department of Clinical and Experimental Medicine, Linköping University, Linköping, Sweden; University of North Carolina at Chapel Hill School of Medicine, UNITED STATES

## Abstract

**Objectives:**

Among patients who underwent primary surgery for non-small cell lung cancer (NSCLC), recurrent disease is frequent and cannot be accurately predicted solely from TNM stage and histopathological features. The aim of this study was to examine the association of tumor markers in pre-operative serum with recurrent disease.

**Material and methods:**

Blood samples were collected prior to lung cancer surgery from 107 patients with stage I-III lung adenocarcinoma surgically treated at Lund University hospital, Lund, Sweden, between 2005 and 2011. The serum tumor markers Carcinoembryonic antigen (CEA), Neuron-specific enolase (NSE), Cancer antigen 125 (CA 125), Human epididymis protein 4 (HE4) and Carbohydrate antigen (CA 19–9) were analyzed retrospectively and clinical follow-up data were collected from patient charts. Forty (37%) patients were diagnosed with recurrent disease.

**Results:**

Sixty-eight (64%) patients had at least one elevated tumor marker prior to surgery. In analysis of disease-free survival (DFS), CA 125 and/or CA 19–9 were significantly associated with recurrent disease adjusted to stage and adjuvant treatment (hazard ratio 2.8, 95% confidence interval 1.4–5.7, p = 0.006).

**Conclusion:**

High pre-operative serum CA 19–9 and/or CA 125 might indicate an increased incidence of recurrent disease in resectable lung adenocarcinomas.

## Introduction

Lung cancer is the most common cause of cancer-related death among men and the second most common cancer-related death among women worldwide [1]. For about 25% of the NSCLC patients, the tumor is diagnosed at an operable stage. The tumor-node-metastasis (TNM) staging system currently represents the best prognostic factor for NSCLC patients in clinical use. However, despite a low TNM stage the risk of recurrence after curatively intended surgery remains high [2]. Tumor markers are easily measured in serum and could thus serve as a convenient complementary method both for diagnostic purposes and for assessment of the risk of metastases. The usefulness of different tumor markers in lung cancer diagnostics, prognostics and disease monitoring has been studied intensely, but often with conflicting results. We have investigated the prognostic role in lung cancer of five tumor markers (CEA, CA 125, HE4, NSE, and CA 19–9), all of which are established in management of different cancers other than lung cancer as described below. We investigated if these tumor markers, already available for clinical analysis and use, could be applicable in lung cancer.

Carcinoembryonic antigen (CEA) is a glycoprotein produced during embryonal and fetal development. In adults it is produced in low amounts in the gastrointestinal tract, the pancreas and liver. Elevated CEA in cancer is hypothesized to be caused by a loss of repression of CEA-encoding genes [3]. CEA is frequently used as a tumor marker in colon carcinoma [4]. In lung cancer, the use of CEA has been reported for differential diagnosis of malignant lung tumor, monitoring of therapy in advanced stages of disease and for detection of recurrent disease [3]. Several studies have suggested CEA as a prognostic marker in non-small cell lung cancer (NSCLC) but results are conflicting [5].

Cancer antigen 125 (CA 125) is a glycoprotein produced in fetal tissue but also in mesothelial cells in adults. It has been extensively studied as a tumor marker for screening and management of ovarian cancer [6, 7]. There are also reports of CA 125 as a marker for worse prognosis in lung cancer [3, 8, 9].

Human epididymis protein 4 (HE4) is a protein expressed in tissues such as genital tract and respiratory epithelium. Overexpression of the protein has been detected in ovarian cancer but also in lung adenocarcinoma and other cancers [10]. It has been suggested as a tumor marker useful in diagnosing ovarian cancer, especially in premenopausal women [7]. In lung cancer it has been suggested as a potential diagnostic [11, 12] and prognostic marker [12, 13].

Neuron-specific enolase (NSE) is a glycolytic neurospecific isoenzyme. It is found in tumors of neural and neuroectodermal origin such as small cell lung cancer (SCLC) and neuroblastoma. It has been reported both as elevated at time of diagnosis and suggested to be useful in follow-up in patients with SCLC. NSE is also found in erythrocytes, plasma cells and platelets and may be released to serum due to hemolysis in the procedure of venipuncture [14]. In patients with NSCLC, NSE has been suggested as a prognostic marker [15] and some studies have presented an association between increased NSE and shorter survival in *EGFR*-mutated NSCLC treated with tyrosine kinase inhibitors (TKI´s) [16, 17].

Carbohydrate antigen (CA 19–9) is used in management of pancreatic tumors[18]. It has also been studied in lung cancer. CA 19–9 in bronchoalveolar lavage fluid, but not in serum, has been identified as a potential diagnostic marker of lung cancer in a study by Ghosh et al. [19]. Ma et al. [20] studied the prognostic value of CA 19–9, NSE, Squamous cell carcinoma antigen (SCC) and Cytokeratin 19 fragment (CYFRA21.1) in serum in stage I NSCLC and showed that CYFRA21.1, but none of the other tumor markers, might be a prognostic marker.

The aim of this study was to investigate the prognostic association of these tumor markers, measured pre-operatively, in a cohort of patients with surgically treated lung adenocarcinoma.

## Material and methods

### Patients

All patients in this study were described previously, as part of the Southern Swedish Lung Cancer Study [21]. The patients were treated surgically at the Lund University hospital between 2005 and 2011. Histology and staging were reviewed by a thoracic pathologist (HB) in terms of Union for International Cancer Control (UICC) 7^th^ edition and WHO classification [22, 23]. Mutation status for epidermal growth factor receptor (*EGFR*) and Kirsten rat sarcoma viral oncogene homolog (*KRAS*) was determined using next generation sequencing (NGS) as previously described [24]. Within the Southern Swedish Lung Cancer Study cohort, all cases of adenocarcinoma histology with a pre-operative blood sample available (n = 107) were selected for the present investigation. None of the patients had received neoadjuvant treatment. Information about treatment and metastatic disease (local or distant) were collected from medical records. Date of recurrent disease was set to date of histological examination or date of radiological examination. Patients diagnosed with a suspected second primary lung cancer (of any histology) were censored at the time the suspected second lung cancer was diagnosed (n = 7) since it would not be possible to distinguish if eventual later recurrences originated from the first or second lung cancer.

When medical records revealed uncertainties regarding potential metastases or if a metastasis might be derived from another primary tumor the cases were censored at the time of the event (n = 5).

Patient-related variables were age, sex and smoking history.

The study was approved by the Ethical Committee at Lund University (762/2004).

### Tumor marker measurement

Blood samples were obtained one to six days prior to surgery or earlier the same day as surgery (except for one case where the available sample was from one month prior to the, delayed, operation). Samples were stored at minus 80 degrees Celsius. Serum was obtained for analysis of CEA, CA 125, CA19-9, NSE and HE4. Tumor marker levels were analyzed with ElectroChemiLuminiscence Immunoassay at the Division of Clinical Chemistry and Pharmacology, Department of Laboratory Medicine, University Hospital, Lund, Sweden.

According to the manufacturers, cutoff values were as follows: CEA<5μg/L, CA 19–9<35kE/L, CA 125 < 35KE/L, NSE<17μg/L, HE4 premenopausal women <92pmol/L and postmenopausal women <121 pmol/L. Female patients >50 years were assumed to be post-menopausal. Cutoff for male patients were not defined and was set to <121pmol/L.

### Statistical analyses

Statistical analyses were conducted using SPSS statistics version 22. Since the tumor markers and the patients’ age did not have a normal distribution (data not shown) nonparametric statistical methods were used and data presented with median and interquartile range (IQR). Analysis of association between categorical variables was performed with the chi-square test or Fisher’s exact test and linear by linear if an ordered category was analyzed. Mann-Whitney’s test was used to compare distributions of continuous variables over categorical variables and Jonkheere-Terpstra test was used to compare distributions of continuous variables over ordered groups. Disease-free survival (DFS) was estimated by the Kaplan-Meier curves and two-sided log-rank tests or log rank trend test were used to compare survival curves. Cox regression was used to estimate the effects of prognostic variables on DFS. A p-value of less than 0.05 was considered statistically significant. NSE was missing in 35 cases (see results) and therefore analyses were performed both with and without NSE included.

## Results

### Patient characteristics

Patient characteristics are summarized in Table 1. Median survival time for those patients alive without a relapse, suspected second primary lung cancer or uncertain metastases at last date of follow-up was 86 months (range 55 to 124 months).

**Table 1:** Patient characteristics.

N = 107		Median (IQR)	Patients (%)
**Gender**	Male		34 (32)
	Female		73 (68)
**Age (years)**		68 (61–75)	
**Smoking history**	Ever/current smoker		91 (85)
	Never smoker		16 (15)
**Stage**	I		56 (52)
	II		31 (29)
	III		20 (19)
*EGFR* mutation*	Yes		13 (12)
	No		88 (82)
	No data		6 (6)
*KRAS* mutation*	Yes		33 (31)
	No		69 (64)
	No data		6 (6)
**Adjuvant treatment (chemotherapy or both chemotherapy and radiotherapy)**	Yes		49 (46)
	No		58 (54)
**Recurrent disease (distant and/or local)**	Yes		40 (37)
		Identified with radiology and cytology/pathology	15 (14)
		Identified with radiology	25 (23)
	No		55 (51)
	Second primary lung cancer **		7 (7)
	Uncertain***		5 (5)

*One tumor hade one *EGFR* mutation and one *KRAS* mutation.

**Defined as none recurrent disease. Cases were censored at the time of diagnosis of second primary lung cancer.

***A suspected metastasis, that has not been confirmed clinically or by histopathology, or a metastasis confirmed or suspected to origin from another primary cancer. Defined as no recurrent disease and the cases were censored at the time of the event.

History of other cancer than lung cancer was collected from the Swedish Cancer Register in the Southern Swedish Healthcare Region. Register data was missing for two patients, but no other cancer was mentioned in their patient records. Patients with another primary malignancy diagnosed within a year from lung cancer surgery, or with a chronic malignancy, are described in S1 Table.

Recurrent disease was diagnosed in 40 patients (37%). However, in one case, liver metastasis that was detected a few weeks after surgery could be visualized also pre-surgery, but was wrongly presumed benign. First site(s) of metastatic disease were thorax (14 patients), CNS (12 patients), skeleton (3 patients), thorax and skeleton (5 patients), liver (2 patients), CNS and thorax (1 patient), thorax, skeleton and CNS (1 patient), CNS and skeleton (1 patient) and kidney and thorax (1 patient). Clinicopathological data and tumor marker data are presented in detail in S2 Table for each patient.

### Elevated tumor markers and level of tumor markers

Analyses of CEA, CA 125, CA 19–9 and HE4 were performed successfully in all pre-operative serum samples (n = 107), whereas analysis of NSE revealed hemolysis in 35 pre-operative samples. Sixty-eight patients (64%) had at least one elevated tumor marker prior to surgery. Distribution of overall positive tumor markers is summarized in Table 2.

**Table 2:** Frequency of patients with 0–5 elevated tumor markers and 0–4 elevated tumor markers (NSE excluded).

No of elevated tumor marker(s) (0–5)	Frequency n (%)	No of elevated tumor markers (0–4) when NSE was excluded	Frequency n (%)
0	39 (36.4%)	0	42 (39.3)
1	42 (39.3%)	1	43 (40.2)
2	20 (18.7%)	2	16 (15.0)
3	4 (3.7%)	3	5 (4.7)
4	1 (0.9%)	4	1 (0.9)
5	1 (0.9%)		

Distribution of specific elevated tumor markers and median and interquartile range (IQR) for each tumor marker are shown in Table 3. There was a tendency of increasing number of positive tumor markers with higher stage (p = 0.13, linear by linear).

**Table 3:** Frequency of positive tumor markers and median and IQR for each tumor marker.

Tumor marker (patients with pre-operative plasma sample)	Frequency of positive tumor marker (%)	Median (IQR)
NSE (n = 72)	9 (12.5)	13.2 (11.0–16.1)
HE4 (n = 107)	25 (23.4)	91.0 (75.0–118)
CA 125 (n = 107)	10 (9.3)	13.0 (9.7–23.0)
CA 19–9 (n = 107)	10 (9.3)	10.3 (6.9–18.7)
CEA (n = 107)	49 (45.8)	4.0 (2.7–9.5)

Jonkheere-Terpstra test revealed a significant difference in tumor marker level for CA 125 (p = 0.008 and p-values from 0.6 to 0.1 for the other four tumor markers) between stages, with higher tumor marker levels in higher stages.

No relationship between discrete categorization of tumor marker levels of any of the tumor markers and *EGFR* or *KRAS* mutation status was found using chi-square test or Fisher’s exact test. The results remained statistically non- significant even when excluding three rare mutations with more unsure clinical relevance (two P848L and one M766I).

Mann-Whitney’s test revealed a significant difference in measured tumor marker level for CA 125 between tumors with *EGFR* mutation and those without (mean rank 54.28 for *EGFR* mutation negative and 28.77 for the *EGFR* mutation positive, p = 0.003) but not for the other tumor markers (p-values from 0.1 to 0.8). When excluding the three rare *EGFR* mutations, there was a significant difference in tumor marker level for CA 125 (mean rank 53.77 for *EGFR* negative and 25.77 for *EGFR* positive, p = 0.004) and HE4 (mean rank 53 for *EGFR* negative and 32.8 for *EGFR* positive, p = 0.04) but not for the other tumor markers (p-values from 0.4 to 0.9). CA 125 level did also significantly differ with *KRAS* mutation status (Mann-Whitney’s test, mean rank 46.41 for *KRAS* mutation negative and mean rank 60.89 for *KRAS* mutation positive, p = 0.02) but not for the other tumor markers (Mann-Whitney’s test, p-values from 0.1 to 0.9).

### Tumor markers and recurrent disease

**Fig 1:**
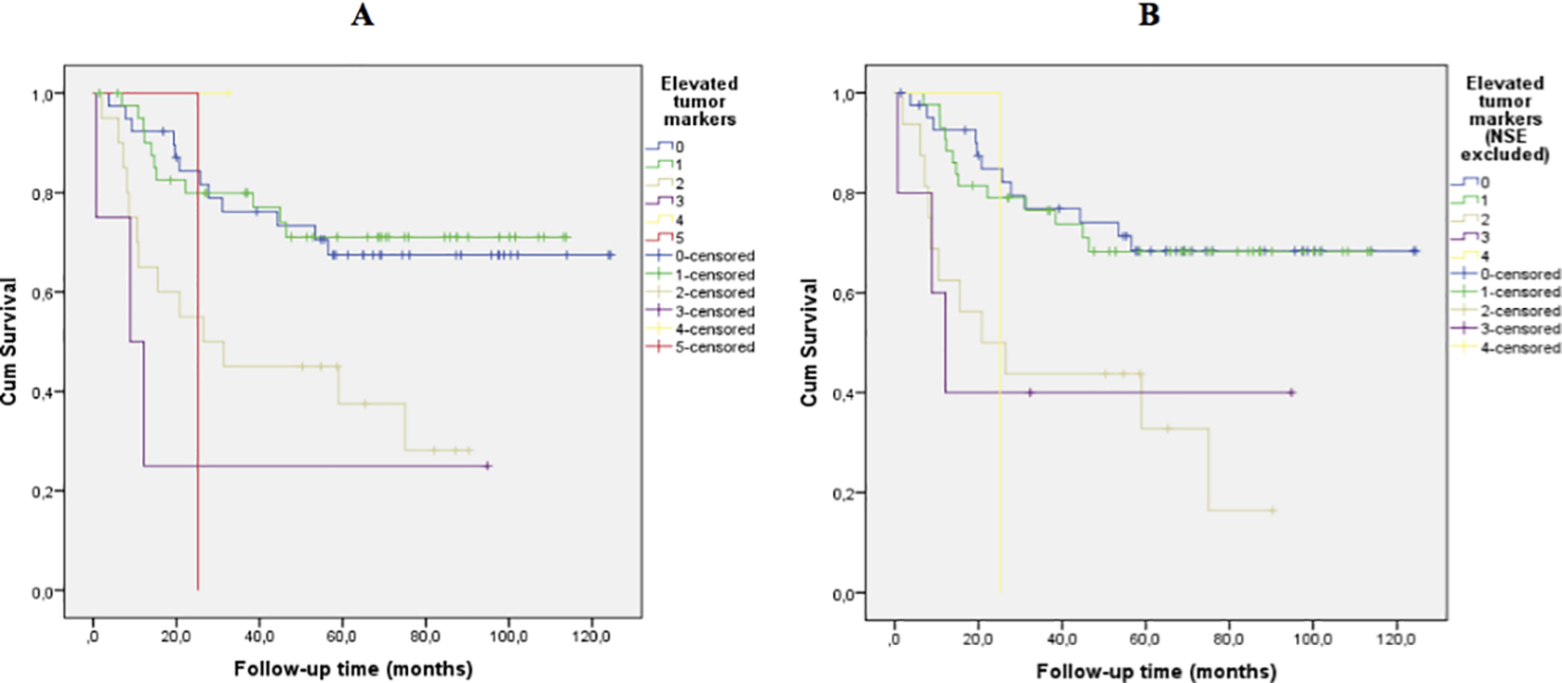
Disease-free survival according to number of elevated tumor markers. (A) All tumor markers included. Log rank trend test p = 0.003 (B) NSE excluded. Log rank trend test p = 0.001.

Survival analysis according to number of elevated tumor markers using DFS as endpoint showed an association of worse prognosis with increasing number of positive tumor markers at time of diagnosis (Fig 1).

**Fig 2:**
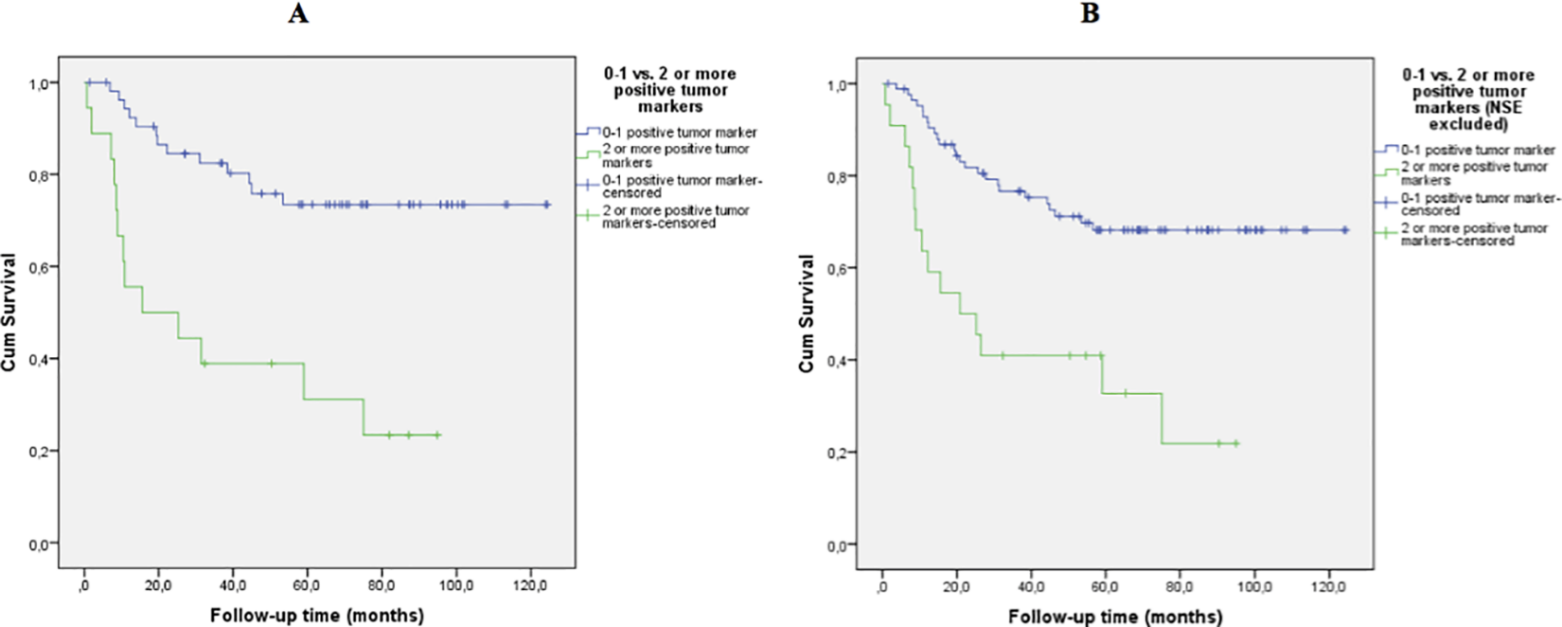
Disease-free survival for cases with none or one positive tumor marker vs cases with two or more positive tumor markers at time of diagnosis. (A) All tumor markers included. Log rank test p< 0.001. (B) NSE excluded. Log rank test p<0.001.

To further analyze this finding we first investigated if DFS differed if the patients had zero compared to at least one positive tumor marker. A non-significant trend of poorer outcome was observed for the latter patient group (log rank test p = 0.18with all tumor markers included and p = 0.14 when NSE was excluded). However, one positive tumor marker compared to zero positive tumor markers was not associated with worse prognosis (log rank test p = 0.86 and p = 0.87 if NSE was excluded). Next, we investigated if there was a difference in DFS between the group of patients with a maximum of 1 positive tumor marker compared to the patients with at least two positive tumor markers (Fig 2), and found a significantly lower DFS for patients with ≥2 elevated tumor markers pre-operative (log rank test p<0.001). Two positive tumor markers compared to one was significantly associated with worse prognosis, log rank test p = 0.003 with and without NSE included.

In log rank tests, all five tumor markers were assumed to have equal importance. To define the importance of each tumor marker cox regression analyses were performed. Association of elevated tumor marker and DFS was evaluated in univariable cox regression for each tumor marker and for age, sex, stage, adjuvant treatment and mutation status (Table 4). As expected, stage was a significant prognostic variable in univariable analysis (although no statistically significant difference between stage II and III was observed). Adjuvant treatment was also defined as a prognostic variable in univariable analysis, probably due to its association with higher stage. Among the tumor markers, positive CA 125 and CA 19–9 displayed a significant association with recurrent disease in univariable analyses and were subsequently analyzed in a multivariable cox regression with stage and adjuvant treatment as covariates (Table 5).

**Table 4:** Univariable cox regression for disease-free survival.

Recurrent disease		
Variable	HR (95% CI)	p-value
CA125	3.7 (1.7–8.1)	0.001
CEA	1.6 (0.8–2.9)	0.2
CA19-9	3.2 (1.4–7.2)	0.006
HE4	1.4 (0.7–2.8)	0.3
NSE	1.1 (0.3–3.8)	0.8
Stage 2 vs 1	3.5 (1.6–7.4)	0.001
Stage 3 vs 1	4.2 (1.8–9.7)	0.001
Sex (male vs. female)	1.6 (0.8–3.0)	0.2
Age	1.0 (0.95–1.0)	0.4
Adjuvant treatment (yes vs. no)	2.4 (1.3–4.6)	0.008
EGFR mutation (yes vs. no)	1.0 (0.4–2.7)	0.9
KRAS mutation (yes vs. no)	1.0 (0.5–2.0)	1.0

**Table 5:** Multivariable cox regression for disease-free survival.

	Recurrent disease	
Variable	HR (95% CI)	p-value
CA125	2.4 (1.1–5.5)	0.04
CA19-9	2.5 (1.0–5.8)	0.04
Stage 2 vs 1	2.9 (1.2–7.3)	0.02
Stage 3 vs 1	3.9 (1.4–10.7)	0.008
Adjuvant treatment (yes vs.no)	0.9 (0.4–2.1)	0.8

With all five tumor markers in a multivariable cox regression analyzing the markers’ impact on DFS, CA 19.9 (HR = 3.3 95% CI = 1.2–9.4 p = 0.02 and when NSE was excluded: HR = 2.5 95%CI 1.0–6.3 p = 0.048) and CA 125 (HR = 6.1 95% CI = 2.4–15.6 p<0.001 and when NSE was excluded: HR = 3.0 95% CI 1.3–6.9 p = 0.008) remained as the tumor markers associated with higher incidence of recurrent disease. P-values for the other three markers ranged from 0.4 (CEA) to 0.6 (HE4) and was 0.5 (CEA) and 0.9 (HE4) respectively when NSE was excluded. In multivariable analyses (Table 5), CA 19–9 and CA 125 remained a significant negative prognostic variable when adjusted to stage and adjuvant treatment. Patients with a positive CA 19–9 and/or CA 125 (18 patients, two with both positive CA 19–9 and CA 125) had a worse prognosis compared to patients with neither positive CA 19–9 nor CA 125 adjusted to stage and adjuvant treatment (HR = 2.8, 95% CI = 1.3–5.7, p = 0.006) Among the eight patients with positive CA 19–9, five were diagnosed with recurrence in thorax, two with a second primary lung adenocarcinoma (censored at time of diagnosis of the second primary lung cancer) and one patient was not diagnosed with metastatic disease during our follow-up. Two of the patients with positive CA 125 were diagnosed with metastases in thorax, four patients with metastases in thorax and/or distant metastases and two patients were not diagnosed with recurrence during our follow-up. Two patients had positive CA 19–9 and CA 125, one was diagnosed with metastatic disease in thorax and one with distant metastasis.

## Discussion

Many patients with lung cancer are diagnosed in a late disease stage, largely explaining the unfavorable prognosis. However, even among patients with early stage, surgically treated disease, a considerable proportion of patients are later diagnosed with local, regional or distant metastases. Tumor markers measured in serum could be a tool for identifying patients with high risk of recurrent disease.

In the current study, we studied the diagnostic and prognostic value of five tumor markers available in clinical practice. The day before surgery, 68 (64%) patients with stage I-III lung adenocarcinoma had one or more positive tumor marker(s). Thus, this combination of markers shows positivity in too few cases to be useful in early diagnostics/screening for lung cancer or for differential diagnosis between lung cancer and other cancer types.

However, we found that even in this relatively small cohort two of the five studied tumor markers seemed to carry prognostic information. CA 125 and CA 19–9 were both associated with an increased incidence of local and/or distant metastases in univariable analyses. Stage is a well-known prognostic factor in lung cancer and CA 19–9 and CA 125 remained significant prognostic variables (HR 2.5 and 2.4, respectively) in subsequent multivariable analyses with stage and adjuvant therapy as covariates. However, due to the size of the cohort and the relatively few events of positive tumor markers results we acknowledge that results should be interpreted carefully, and that larger, prospective studies are needed to confirm the prognostic associations. Moreover, it is clear that patients without increased levels of tumor markers pre-operative could still develop later metastases and all patients with increased tumor markers were not diagnosed with metastases during our follow-up time. Still, our results points towards an increased incidence of recurrence for patients with a positive pre-operative CA 19–9 and/or CA 125.

In the literature, we could only find few reports of CA19-9 and its potential use in lung cancer, whereas pre-operative CA 125 measured in serum in operable patients has been suggested as a prognostic factor in NSCLC in some studies [25]. However, there are also conflicting results for CA 125 in the literature. For instance, Ma et al. investigated the prognostic values of CA 125, CA 19–9, NSE, CYFRA21.1 and SCC in samples one week before surgery in stage I NSCLC patients and found that CYFRA21.1 was an independent prognostic factor regarding overall survival but the other tumor markers were not [20]. In their study the positive rate of CA 125 and CA19-9 was low, 5% and 4% respectively. In our study, higher positive rates were observed, 9.3% for both CA 125 and CA 19–9, potentially explained by the fact that our study included tumors of stages I-IIIA, while the former study focused only on stage I tumors.

In our study, with adenocarcinoma as the only histological subtype, results revealed a significantly shorter DFS in the group with high CA 125 in univariable analysis. This result is similar to results reported by Yu et al. who enrolled 481 patients with operable NSCLC in a study to investigate the prognostic value of NSE and CA 125, finding that both markers could be useful in predicting prognosis [15] although conflicting results have been reported [26]. In the current study we could not reproduce the results for NSE, possibly due to low sample numbers.

The present study has some limitations. The study size is relatively small and analysis of NSE is difficult to interpret due to low sample numbers. Furthermore, the majority of patients included are females (68% compared to 54% females among all lung adenocarcinoma patients in Sweden 2002–2015 [27]) and smokers (85%). Future studies are indeed needed to investigate potential differences between subgroups of lung cancer patients. Moreover, in future studies, cut-off values for the different tumor markers need to be derived for lung cancer specifically. A larger prospective study could potentially also identify combinations of tumor markers useful in the management of lung cancer patients. Still, our present results suggest that pre-operative serum CA 19–9 and/or CA 125 might correlate with worse DFS in surgically treated lung adenocarcinoma.

## Conclusions

Our results indicate that elevated pre-operative serum tumor markers might be associated with an increased incidence of recurrent disease. Specifically, CA 19–9 and CA 125 were identified as potential informative pre-operative markers according to established cutoff values.

## Supporting information

S1 Table Patients with chronic cancer or cancers other than lung cancer diagnosed within one year from lung cancer surgery. (DOCX)

S2 Table Patient and tumor marker data. (XLSX)

## References

[1] Torre LA, Bray F, Siegel RL, Ferlay J, Lortet-Tieulent J, Jemal A. Global cancer statistics, 2012. CA: A Cancer Journal for Clinicians. 2015;65(2):87–108. doi: 10.3322/caac.21262 25651787 10.3322/caac.21262

[2] Kelsey CR, Marks LB, Hollis D, Hubbs JL, Ready NE, D'Amico TA, et al Local recurrence after surgery for early stage lung cancer. Cancer. 2009;115(22):5218–27. doi: 10.1002/cncr.24625 19672942 10.1002/cncr.24625

[3] Molina R, Holdenrieder S, Auge JM, Schalhorn A, Hatz R, Stieber P. Diagnostic relevance of circulating biomarkers in patients with lung cancer. Cancer Biomarkers. 2009;6(3):163–78. doi: 10.3233/CBM-2009-0127 20660962 10.3233/CBM-2009-0127 PMC12922860

[4] Figueredo A, Rumble RB, Maroun J, Earle CC, Cummings B, McLeod R, et al Follow-up of patients with curatively resected colorectal cancer: a practice guideline. BMC Cancer. 2003;3(1):1–13. doi: 10.1186/1471-2407-3-26 14529575 10.1186/1471-2407-3-26 PMC270033

[5] Crosbie PAJ, Shah R, Summers Y, Dive C, Blackhall F. Prognostic and predictive biomarkers in early stage NSCLC: CTCs and serum/plasma markers. Translational Lung Cancer Research. 2013;2(5):382–97. doi: 10.3978/j.issn.2218-6751.2013.09.02 25806257 10.3978/j.issn.2218-6751.2013.09.02 PMC4367728

[6] Doubeni CA, Doubeni AR, Myers AE. Diagnosis and Management of Ovarian Cancer. American family physician. 2016;93(11):937–44. Epub 2016/06/10. . 27281838

[7] Sölétormos G, Duffy MJ, Hassan SOA, Verheijen RHM, Tholander B, Bast RC, et al Clinical Use of Cancer Biomarkers in Epithelial Ovarian Cancer: Updated Guidelines from the European Group on Tumor Markers (EGTM). International journal of gynecological cancer: official journal of the International Gynecological Cancer Society. 2016;26(1):43–51. doi: 10.1097/IGC.0000000000000586 PMID: PMC4679342. 26588231 10.1097/IGC.0000000000000586 PMC4679342

[8] Cedrés S, Nuñez I, Longo M, Martinez P, Checa E, Torrejón D, et al Serum Tumor Markers CEA, CYFRA21-1, and CA-125 Are Associated With Worse Prognosis In Advanced Non–Small-Cell Lung Cancer (NSCLC). Clinical Lung Cancer. 2011;12(3):172–9. doi: 10.1016/j.cllc.2011.03.019 21663860 10.1016/j.cllc.2011.03.019

[9] Gaspar MJ, Diez M, Rodriguez A, Ratia T, Martin Duce A, Galvan M, et al Clinical value of CEA and CA125 regarding relapse and metastasis in resectable non-small cell lung cancer. Anticancer Res. 2003;23(4):3427–32. Epub 2003/08/21. . 12926084

[10] Galgano MT, Hampton GM, Frierson HF Jr., Comprehensive analysis of HE4 expression in normal and malignant human tissues. Modern pathology: an official journal of the United States and Canadian Academy of Pathology, Inc. 2006;19(6):847–53. Epub 2006/04/12. doi: 10.1038/modpathol.3800612 . 16607372 10.1038/modpathol.3800612

[11] Zeng Q, Liu M, Zhou N, Liu L, Song X. Serum human epididymis protein 4 (HE4) may be a better tumor marker in early lung cancer. Clinica Chimica Acta. 2016;455:102–6. http://dx.doi.org/10.1016/j.cca.2016.02.002. 10.1016/j.cca.2016.02.002 26851650

[12] Iwahori K, Suzuki H, Kishi Y, Fujii Y, Uehara R, Okamoto N, et al Serum HE4 as a diagnostic and prognostic marker for lung cancer. Tumor Biol. 2012;33(4):1141–9. doi: 10.1007/s13277-012-0356-9 22373583 10.1007/s13277-012-0356-9

[13] Lamy P-J, Plassot C, Pujol J-L. Serum HE4: An Independent Prognostic Factor in Non-Small Cell Lung Cancer. PloS one. 2015;10(6):e0128836 doi: 10.1371/journal.pone.0128836 PMID: PMC4452338. 26030627 10.1371/journal.pone.0128836 PMC4452338

[14] Harmsma M, Schutte B, Ramaekers FCS. Serum markers in small cell lung cancer: Opportunities for improvement. Biochimica et Biophysica Acta (BBA)—Reviews on Cancer. 2013;1836(2):255–72. http://dx.doi.org/10.1016/j.bbcan.2013.06.002. 23796706 10.1016/j.bbcan.2013.06.002

[15] Yu D, Du K, Liu T, Chen G. Prognostic value of tumor markers, NSE, CA125 and SCC, in operable NSCLC Patients. Int J Mol Sci. 2013;14(6):11145–56. Epub 2013/05/29. doi: 10.3390/ijms140611145 . 23712355 10.3390/ijms140611145 PMC3709724

[16] Suh KJ, Keam B, Kim M, Park YS, Kim TM, Jeon YK, et al Serum Neuron-Specific Enolase Levels Predict the Efficacy of First-Line Epidermal Growth Factor Receptor (EGFR) Tyrosine Kinase Inhibitors in Patients With Non-Small Cell Lung Cancer Harboring EGFR Mutations. Clinical Lung Cancer. http://dx.doi.org/10.1016/j.cllc.2015.11.012. 10.1016/j.cllc.2015.11.012 26719155

[17] Inomata M, Hayashi R, Yamamoto A, Tokui K, Taka C, Okazawa S, et al Plasma neuron-specific enolase level as a prognostic marker in patients with non-small cell lung cancer receiving gefitinib. Molecular and Clinical Oncology. 2015;3(4):802–6. doi: 10.3892/mco.2015.568 PMID: PMC4486842. 26171184 10.3892/mco.2015.568 PMC4486842

[18] Goonetilleke KS, Siriwardena AK. Systematic review of carbohydrate antigen (CA 19–9) as a biochemical marker in the diagnosis of pancreatic cancer. European Journal of Surgical Oncology (EJSO). 2007;33(3):266–70. http://dx.doi.org/10.1016/j.ejso.2006.10.004. 17097848 10.1016/j.ejso.2006.10.004

[19] Ghosh I, Bhattacharjee D, Das AK, Chakrabarti G, Dasgupta A, Dey SK. Diagnostic Role of Tumour Markers CEA, CA15-3, CA19-9 and CA125 in Lung Cancer. Indian journal of clinical biochemistry: IJCB. 2013;28(1):24–9. Epub 2014/01/02. doi: 10.1007/s12291-012-0257-0 . 24381417 10.1007/s12291-012-0257-0 PMC3547445

[20] Ma S, Shen L, Qian N, Chen K. The prognostic values of CA125, CA19.9, NSE, AND SCC for stage I NSCLC are limited. Cancer Biomarkers. 2011;10(3):155–62. doi: 10.3233/CBM-2012-0246 22674301 10.3233/CBM-2012-0246 PMC13016267

[21] Brunnström H, Johansson L, Jirström K, Jönsson M, Jönsson P, Planck M. Immunohistochemistry in the differential diagnostics of primary lung cancer: an investigation within the Southern Swedish Lung Cancer Study. American journal of clinical pathology. 2013;140(1):37–46. doi: 10.1309/AJCP50RDXSCSBTBO 23765532 10.1309/AJCP50RDXSCSBTBO

[22] Travis WD BE, Brambilla E, Müller-Hermelink HK, Harris CC, eds. WHO Classification of Tumours of the Lung, Pleura, Thymus and Heart. 7^th^ ed. Lyon, France: IARC Press 2004.

[23] Sobin L GM, Wittekind C, eds. International Union Against Cancer (UICC) TNM classification of malignant tumours. 7th ed. Chichester, UK: Wiley-Blackwell; 2009.

[24] Lindquist KE, Karlsson A, Levéen P, Brunnström H, Reuterswärd C, Holm K, et al Clinical framework for next generation sequencing based analysis of treatment predictive mutations and multiplexed gene fusion detection in non-small cell lung cancer. Oncotarget. 2017;8(21):34796–810. doi: 10.18632/oncotarget.16276 PMID: PMC5471012. 28415793 10.18632/oncotarget.16276 PMC5471012

[25] Pollán M, Varela G, Torres A, de la Torre M, Ludeña MD, Ortega MD, et al Clinical value of p53, c-erbB-2, CEA and CA125 regarding relapse, metastasis and death in resectable non-small cell lung cancer. International Journal of Cancer. 2003;107(5):781–90. doi: 10.1002/ijc.11472 14566828 10.1002/ijc.11472

[26] Reinmuth N, Brandt B, Semik M, Kunze W-P, Achatzy R, Scheld HH, et al Prognostic impact of Cyfra21-1 and other serum markers in completely resected non-small cell lung cancer. Lung Cancer. 2002;36(3):265–70. http://dx.doi.org/10.1016/S0169-5002(02)00009-0. 12009236 10.1016/s0169-5002(02)00009-0

[27] Landstingens och regionernas nationella samverkansgrupp inom cancersjukvården. Lungcancer Årsrapport från nationella lungcancerregistret (NCLCR). 2015. Available from: http://www.cancercentrum.se/globalassets/cancerdiagnoser/lunga-ochlungsack/kvalitetsregister/rapport/nlcr_tapport_tom2015_korr170111.pdf.

