# Supplementary material for: CA 19-9 and CA 125 as potential predictors of disease recurrence in resectable lung adenocarcinoma
Source: PLoS One. 2017 Oct 19;12(10):e0186284. doi: 10.1371/journal.pone.0186284 (PMC5648153; doi:10.1371/journal.pone.0186284)
Supplement: S1 Table — (DOCX) [file pone.0186284.s001.docx]

**S1 Table**Patients with chronic cancer or cancers other than lung cancer diagnosed within one year from lung cancer surgery

| Patient no | Other cancer(s) than lung cancer |
| --- | --- |
| 1 | Rectal carcinoma (T1) treated surgically. Lung tumor was identified through pre-operative radiological examination. |
| 2 | Squamous cell carcinoma in larynx revealed during bronchoscopy when investigating the lung tumor. |
| 3 | Surgical treatment of prostate cancer 8 years before lung cancer diagnosis. Recurrent disease (increased PSA), treated with hormone injections 3 years after prostate cancer surgery. |
| 4 | Chronic lymphatic leukemia diagnosed 15 years before lung cancer diagnose. |
| 5 | Breast cancer diagnosis and lung cancer surgery the same year. The lung tumor was suspected to be a metastasis but histological analysis revealed a primary lung tumor. Hormonal therapy for breast cancer. |
| 6 | Chronic lymphocytic leukemia diagnosed within a year prior lung cancer surgery. |
| 7 | Relapsing highly differentiated bladder cancer initially diagnosed 8 years before lung cancer diagnosis. One relapse in the year before lung cancer surgery. |
| 8 | A squamous cell carcinoma metastasis in the neck with unknown primary tumor 5 years before lung adenocarcinoma was surgically treated. The diseases are considered two separate conditions by the clinicians. |
| 9 | Lymphoma was discovered during investigation of lung tumor. |
| 10 | Lung cancer surgery the year before nephrectomy was performed because of papillary renal cancer and the patient was also diagnosed with an asymptomatic myeloma. The same year as lung cancer surgery the patient was diagnosed with prostate cancer, transurethral resection. |
| 11 | Breast cancer diagnosis the same year as lung cancer surgery. Hormonal therapy for breast cancer. |
| 12 | Breast cancer surgery same year as lung cancer surgery. Post-operative hormonal therapy for breast cancer. |
| 13 | Squamous cell carcinoma of the lip the year before cancer surgery. |
